# Rhabdomyolysis and Acute Kidney Injury Requiring Dialysis as a Result of Concomitant Use of Atypical Neuroleptics and Synthetic Cannabinoids

**DOI:** 10.1155/2015/235982

**Published:** 2015-10-13

**Authors:** Aiyu Zhao, Maybel Tan, Aung Maung, Moro Salifu, Mary Mallappallil

**Affiliations:** State University of New York Downstate Medical Center, 450 Clarkson Avenue, Brooklyn, NY 11203, USA

## Abstract

The use of synthetic cannabinoids (SCBs) is associated with many severe adverse effects that are not observed with marijuana use. We report a unique case of a patient who developed rhabdomyolysis and acute kidney injury (AKI) requiring dialysis after use of SCBs combined with quetiapine. Causes for the different adverse effects profile between SCBs and marijuana are not defined yet. Cases reported in literature with SCBs use have been associated with reversible AKI characterized by acute tubular necrosis and interstitial nephritis. Recent studies have showed the involvement of cytochromes P450s (CYPs) in biotransformation of SCBs. The use of quetiapine which is a substrate of the CYP3A4 and is excreted (73%) as urine metabolites may worsen the side effect profiles of both quetiapine and K2. SCBs use should be included in the differential diagnosis of AKI and serum Creatinine Phosphokinase (CPK) level should be monitored. Further research is needed to identify the mechanism of SCBs nephrotoxicity.

## 1. Introduction

AKI is the abrupt loss of kidney function, resulting in the retention of urea and other nitrogenous waste products and in the dysregulation of volume and electrolytes [1]. Rhabdomyolysis is characterized by muscle necrosis and the leakage of muscle-cell contents like electrolytes, myoglobin, and sarcoplasmic proteins (CPK, aldolase, lactate dehydrogenase, alanine aminotransferase, and aspartate aminotransferase) into the circulation [2]. AKI is a complication of severe rhabdomyolysis and is seen in about 7–10% of all cases of AKI in the United States. The causes of rhabdomyolysis may be classified as traumatic, nontraumatic exertional, and nontraumatic nonexertional causes [2, 3].

Cannabis is the most commonly used illegal substance in the world [4]. It contains over 400 compounds, including more than 60 cannabinoids. The primary psychoactive cannabinoid is delta-9-tetrahydrocannabinol (THC) [5].

SCBs have multiple brand names most commonly “Spice” or “K2” and many street names such as “Fake Pot” [5]. They are a heterogeneous group of compounds developed to probe the endogenous cannabinoid system (ECS) [4, 5]. SCBs can be divided into 7 major structural groups: naphthoylindoles (JWH-018 and JWH-073), naphthylmethylindoles, naphthoylpyrroles, naphthylmethylindenes, phenylacetylindoles (JWH-250), cyclohexylphenols (CP-47,497), and classical cannabinoids (HU-210). There are no structural similarities in SCBs with THC [5, 6].

Recreational use of SCBs was noticed initially in the early 2000s in Europe. After European and Russian authorities banned SCBs in 2010, the K2 epidemic emerged in the United States [5]. Previously they were referred to as “legal highs” or “herbal highs”; these compounds are now classified as Class I controlled substances by the United States Drug Enforcement Administration [7]. Illicit use remains significant and reports of illness are increasing. In March 2012, 16 cases of AKI after SCBs use were reported in six states [8]. Four cases of oliguric AKI associated with use of SCBs were reported in 2013 [9]. However, rhabdomyolysis was not reported in the above cases. To our knowledge, there is only case report of rhabdomyolysis associated with SCB use in the USA [10].

We are reporting a unique case of a patient with history of Cannabis dependence and paranoid schizophrenia who developed severe rhabdomyolysis and AKI requiring dialysis after use of K2 combined with quetiapine.

## 2. Case Presentation

A 39-year-old African American man from a supervised living facility, with history of paranoid schizophrenia and Cannabis dependence, presented with generalized bodyache, back pain, and weakness. He had been smoking one joint of K2 daily purchased from the street for several years, with increased use in the one week prior to admission. The day prior to admission he took 10 tablets of quetiapine from his roommate with the intention of suicide. Subsequently he felt nauseated and vomited. He noticed that his urine “was darker.” He had a history of paranoid schizophrenia with many failed antipsychotic regimens. In the last 2 years, he had been receiving monthly intramuscular haloperidol decanoate 250 mg and the last injection was 3 weeks prior to admission. He denied history of trauma or injury and denied chest pain, shortness of breath or dizziness or other medications, and supplement or other illicit drugs' use. There was no similar episode in the past.

On examination, he was afebrile, initial blood pressure was 136/87 mmHg with pulse of 111 per minute, and respiratory rate was 18 per minute. There was no orthostatic hypotension. Oxygenation saturation was 100% in room air. He was lethargic but oriented to person, place, and date. His pupils were equal and reactive to light and measured about 3 mm in size. His lungs were clear to auscultation; heart rate was regular with no murmurs; abdomen was soft and there is no tenderness or organomegaly. There was 2+ pitting edema in bilateral lower extremities up to the knees; there was diffuse tenderness upon palpation. Foley catheter was inserted with 50 milliliters of tea color urine returned.

Table 1 shows the daily laboratory values. His creatinine was 1 mg/dL (88.4 *μ*mol/L) in November 2013. Urine microscopy showed muddy brown casts of acute tubular necrosis. Urine myoglobin was strongly positive. FeNa (the fractional excretion of sodium) was 1.8%. Urine toxicity screen for barbiturate, benzodiazepines, cocaine, methadone, and opiates was negative. Urine for cannabinoid was negative as expected with SCBs use. Alcohol and salicylate level was undetectable. HIV (the human immunodeficiency virus), HBV (hepatitis B virus), HCV (hepatitis C virus), EBV (Epstein-Barr virus), anti-DNase B, ANA (antinuclear antibody), P-ANCA (Perinuclear Anti-Neutrophil Cytoplasmic Antibodies), C-ANCA (Cytoplasmic Anti-Neutrophil Cytoplasmic Antibodies), and anti-glomerular basement membrane (anti-GBM) antibody were all negative; complements were within normal limits. ABG revealed pH of 7.29, PCO_2_ of 30.6 mmHg, PO_2_ of 178 mmHg, and bicarbonate of 16 mmol/L, which was consistent with high anion gap metabolic acidosis with respiratory compensation. High anion metabolic acidosis is likely due to AKI, as there was no evidence of lactic acidosis, ketoacidosis, toxic alcohol, acetaminophen, or salicylate ingestion.

Electrocardiogram showed normal sinus rhythm with no PT prolongation. There was no hydronephrosis in renal sonogram.

A diagnosis of AKI secondary to rhabdomyolysis and K2 use was made. His FeNa was more than 1 which is different from typical rhabdomyolysis-induced AKI when FeNa may frequently be less than 1%. The workup for AKI has been unrevealing except for positive urine myoglobin. The additional K2 nephrotoxicity might have explained this finding [11, 12]. The etiology of rhabdomyolysis was thought to be secondary to use of K2 combined with quetiapine. Neuroleptic malignant syndrome was in the differential diagnosis, however, thought to be less likely as the patient had never been febrile with no rigidity and no tremor or signs of autonomic dysfunction.

He was started on aggressive intravenous hydration with normal saline at 200 mL/hr. After 12 hours, no increase in urine output was noticed. The IV fluid rate increased to 500 mL/hour; he remained oliguric with persistent hyperkalemia. Hemodialysis was started on Day 3 of the hospitalization. He was dialyzed for 10 sessions. Urine output started to improve on Day 13 when his 24 hours' urine output was 900 mL. His last dialysis was on Day 14 of hospitalization after which creatinine and CK continued to decrease without dialysis. After 26 days of hospitalization he was discharged back to the supervised living facility; upon discharge, his creatinine was 2.25 mg/dL and CK was 299 IU/L.

With the resolution of oliguria in AKI which is the commonest marker for improvement in renal function, we noted a decrease in serum CPK and decrease in the number of muddy brown casts on sequential urine microscopy [11].

## 3. Discussion

Unlike many other “traditional” drugs of abuse, SCBs appear to have variable and unknown toxicities, including many not seen with marijuana use like tachycardia, hypertension, nausea, vomiting, convulsions, agitation, hallucinations, and psychosis [4, 5]. More recently, separate reports of rhabdomyolysis and kidney failure after SCBs use have been published [4, 5, 7–10, 13]. SCBs use has also been implicated in cases of acute myocardial infarction in three otherwise healthy teenagers. Dependence and withdrawal symptoms associated with chronic use have also been reported [6].

SCBs interact with cannabinoid receptors (CBRs) and elicit cannabimimetic effects similar to THC [6, 14, 15]. The endogenous cannabinoid system (ECS) is widely dispersed through the body. To date, two endogenous cannabinoid receptors, CBR1 and CBR2, are well characterized [6, 14, 15]. CBR1 and CBR2 are G protein-coupled receptors (GPCRs). CBR1 is mainly expressed in the brain and mediates the CNS effects of THC and other cannabinoids. CBR1 also is expressed peripherally in adipocytes and skeletal muscle. Recent studies have showed that ECS affects skeletal muscle oxidation [6]. CBR2 is located primarily on the T cells, B cells, and macrophages and in hematopoietic cells and is involved in regulating immune function. In general, CBR2 activation is immunosuppressive, inhibits production of proinflammatory cytokines, enhances production of anti-inflammatory cytokines, induces apoptosis of immune cells, and suppresses macrophage chemotaxis. Activation of CBR2 is thought to underlie the anti-inflammatory and immunosuppressive effects of marijuana [6, 16].

Causes for the different adverse effects profile between SCBs and marijuana are not defined yet. Four possible mechanisms are postulated [6]. The first two mechanisms are potential differences in pharmacodynamics. First, these effects are probably mediated by actions of SCBs at noncannabinoid receptors. Second, as most SCBs examined to date possess higher potency and efficacy than THC at CBRs it is possible that SCBs, especially taken in the various combinations found in SCB products, achieve levels of CBR1 and CBR2 activation high enough to produce severe, clinically observable physiological and psychological disturbances.

Third, the active components of marijuana and SCBs are likely metabolized differently and thus have distinct pharmacokinetic profiles. Some active metabolites of SBCs likely contribute to the effects by activating CBRs. THC is metabolized by CYP2C9 to a predominant single biologically active metabolite, 11-hydroxy-D9-THC, which then inactivated carboxylation and glucuronidated prior to excretion. In contrast, several major metabolites of SCBs exhibit greater CBR1 affinity, potency, and efficacy than THC, both in vitro and in vivo. And these metabolites also retain in vitro pharmacological activity at CBR2s with greater potency.

The fourth mechanism is that drug-drug interactions, such as synergy, may increase risk of adverse effects as multiple drug use is common with SCBs abuse.

According to a study by Ginsburg et al. in 2012, adverse effects following consumption of SCBs are unlikely due to impurities or residue from the manufacturing process [17]. However, different content of nonpsychoactive substances in the marijuana plant versus SCB products might play a role in the adverse symptoms.

Neuroleptics can cause acute rhabdomyolysis as part of a neuroleptic malignant syndrome (NMS) or via direct toxic effect on myocytes. One suggested mechanism of rhabdomyolysis in the absence of NMS is an increase of skeletal muscle-cell membrane permeability [2, 3]. It is unknown whether rhabdomyolysis is dose or duration dependent when neuroleptics are involved [13]. There has been evidence that endocannabinoid has effect on the skeletal muscle oxidation [18]. But the complete endocannabinoid action on skeletal muscle metabolism is still to be elucidated. If both SCBs and quetiapine have effect on skeletal muscle, the combination of them might have made the injury more severe.

SCB causing AKI is thought to be via acute tubular necrosis which does not present with rhabdomyolysis. Cases reported in literature with K2 use and AKI have been associated with reversible acute kidney injury characterized by acute tubular necrosis and interstitial nephritis [9]. THC is initially metabolized via oxidation by CYP2C9 and CYP3A4 [19, 20]. Recent in vitro metabolism studies, using recombinant CYPs, identified CYP2C9 and CYP1A2 as the primary hepatic P450 isoforms involved in the oxidation of JWH-018 [19, 21]. Other structural groups of SCBs might have involved CYP3A4. In this case the combination with quetiapine which is a substrates of the CYP3A4 system and which is excreted mostly (73%) in the urine as metabolites may worsen the side effect profiles of both quetiapine and K2.

## 4. Conclusion

Complete pharmacological knowledge of synthetic cannabinoids is lacking. The confirmation of SCBs use was hindered by lack of known biomarkers. It is important for the clinician to always take a thorough history and have a complete list of medications the patients are exposed to. SCBs use should be included in the differential diagnosis of AKI and CK level should be monitored. Further research is needed to identify the mechanism of SCBs nephrotoxicity.

## Figures and Tables

**Table 1:** Daily laboratory values.

	CK (IU/L)	Potassium (mmol/L)	BUN (mg/dL)	Creatinine (mg/dL)	AST (IU/L)	ALT (IU/L)	Urine output (mL)	Note
Day 1	>22,000	6.9	68	6.09	3167	964	10	IVF
Day 2	148,643	6.5	83	7.56	2307	724	20	IVF
Day 3	>22,000	6.8	92	9.39	1520	597	0	HD
Day 4	81,620	5.2	84	9.15	973	530	0	HD
Day 5	>22,000	5.6	87	9.69	610	442	0	HD
Day 6	51,030	4.8	75	8.36	523	406	0	HD
Day 7	28,060	4.9	76	8.77	315	331	0	HD
Day 8	16,339	4.4	62	7.79	222	237	50	No HD
Day 9	10,701	5	75	9.7	188	218	100	HD
Day 10	6242	4.4	63	8.87	128	200	150	HD
Day 11	4230	4.8	58	8.75	91	161	200	HD
Day 12	2719	4.9	58	8.61	65	123	300	No HD
Day 13	2199	4.6	64	9.38	57	112	900	HD
Day 14	1835	4.4	57	8.69	51	109	1200	HD

CK: Creatine Kinase, BUN: Blood Urea Nitrogen, AST: Aspartate Transaminase, and ALT: Alanine Transaminase.
